# Spatial architecture of the melanoma immune niche reveals CORO1A as a functional hub for T cell cytotoxicity and immunotherapy synergy

**DOI:** 10.1186/s12967-026-08435-0

**Published:** 2026-06-23

**Authors:** Shuai Zhang, Haoxinai Wang, Jingjing Wu, Pengfei Fan, Yun Liu, Tengxiao Ma, Lei Li

**Affiliations:** 1 Department of Plastic and Cosmetic Surgery, Hainan General Hospital, Hainan Affiliated Hospital of Hainan Medical University, Haikou, Hainan 570311 China; 2 Operative Room, Hainan General Hospital, Hainan Affiliated Hospital of Hainan Medical University, Haikou, Hainan 570311 China

**Keywords:** Spatial transcriptomics, Tumor microenvironment (TME), Melanoma, Immunotherapy, *CORO1A*, Prognostic biomarker

## Abstract

**Background:**

Immune checkpoint blockade (ICB) often fails in cutaneous melanoma due to an incomplete understanding of the spatially organized tumor microenvironment (TME). This study aims to define the spatial architecture of the melanoma TME and identify key molecular regulators that determine anti-tumor immunity and ICB response.

**Methods:**

We employed an integrative analysis of single-cell and spatial transcriptomics to map the TME. We defined spatial domains and deconvoluted their cellular composition. Cell-cell communication and risk modeling were performed. Functional roles of candidate genes were assessed using in vitro co-culture with anti-PD-1 and in vivo knockdown in a humanized mouse model.

**Results:**

We delineated two prognostically significant spatial domains: an Immune region and a Melanocyte region. Multi-omics convergence nominated CORO1A as a key regulator within the immune niche. Its knockdown synergized with anti-PD-1 to suppress tumor growth in vivo, and anti-PD-1 downregulated its expression in vitro. We further identified APP-CD74 and FN1–CD44 as key inter-domain communication axes.

**Conclusion:**

Our study provides a spatially resolved blueprint of the melanoma TME and identifies *CORO1A* as a functional regulator within the immune niche, where its modulation enhances T-cell activity and synergizes with ICB. These findings reveal spatial organization as a critical determinant of immunotherapy efficacy and nominate *CORO1A* as a promising target for combination therapy.

**Graphical Abstract:**

**Figure:**
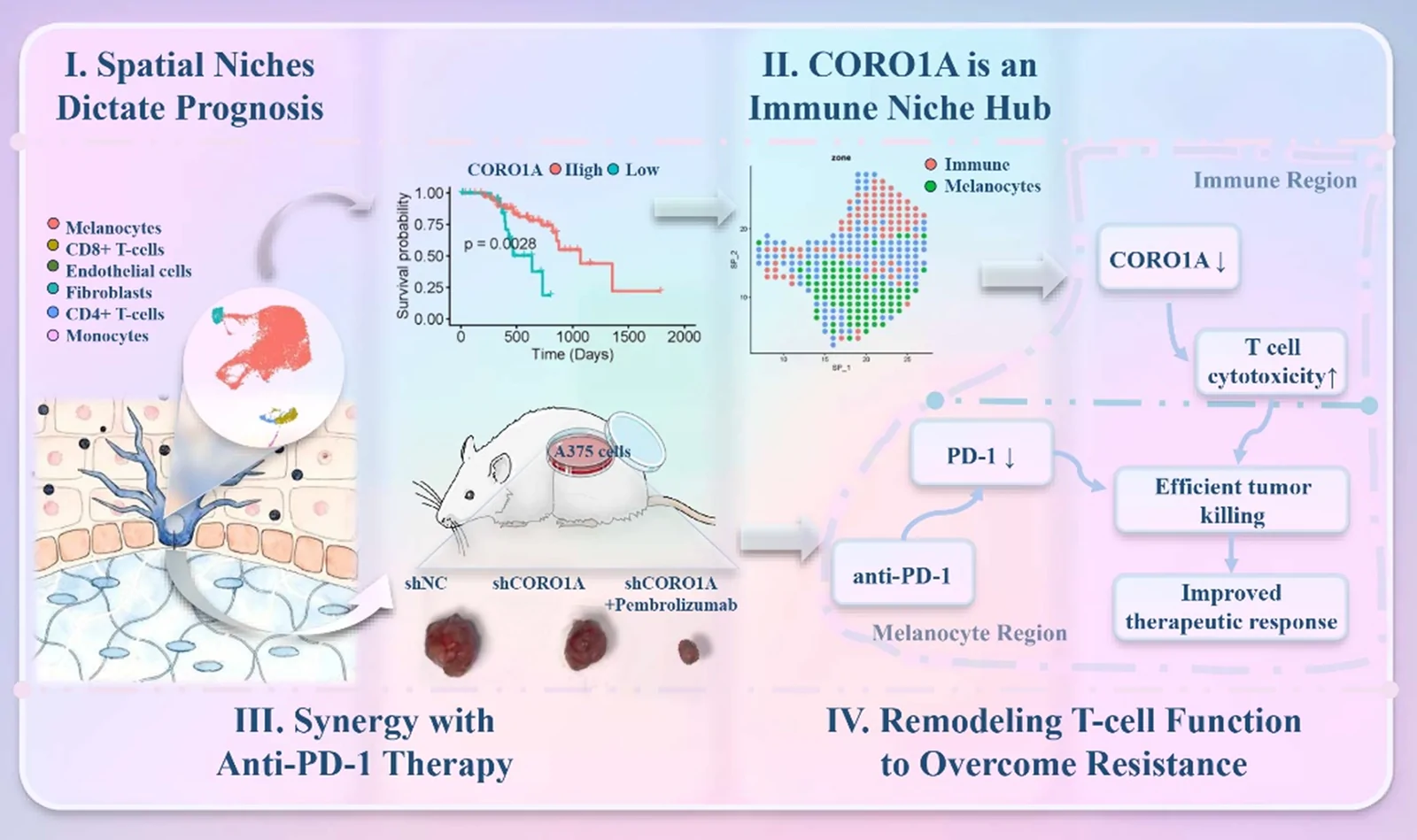

**Supplementary Information:**

The online version contains supplementary material available at https://doi.org/10.1186/s12967-026-08435-0.

## Key messages

**What is already known on this topic:**

Immune checkpoint blockade has transformed melanoma treatment, but resistance remains common. While spatial organization of the tumor microenvironment is recognized as important for immunotherapy response, a systematic mapping of functional spatial domains in human melanoma and their key regulators is lacking.

**What this study adds:**

This study provides a spatially resolved blueprint of the melanoma microenvironment, identifying two prognostically distinct domains. We discovered CORO1A as a key functional regulator within the immune niche that modulates T-cell cytotoxicity and synergizes with anti-PD-1 therapy, and revealed APP-CD74 and FN1–CD44 as critical inter-domain communication axes.

**How this study might affect research, practice or policy:**

Our findings establish spatial organization as a critical determinant of immunotherapy response and nominate CORO1A as a novel therapeutic target. The spatial domain-derived risk models could improve patient stratification and guide personalized combination therapies in clinical practice.

**Supplementary Information:**

The online version contains supplementary material available at https://doi.org/10.1186/s12967-026-08435-0.

## Introduction

Cutaneous melanoma represents the most aggressive form of skin cancer. Despite the transformative impact of immune checkpoint blockade (ICB) targeting axes such as PD-1/PD-L1, the majority of patients still develop resistance, underscoring a fundamental gap in our understanding of the tumor microenvironment (TME) [1–3]. Emerging evidence from other malignancies highlights diverse mechanisms—such as VEGF-A signaling, genetic polymorphisms, and nanoparticle-mediated effects—that contribute to treatment outcomes, further emphasizing the complexity of therapeutic resistance [4–6]. While ICB efficacy depends on CD8^+^ T cell infiltration and function [7, 8], their mere presence is often insufficient for tumor elimination [9–11]. This paradox highlights a critical, unresolved challenge: defining the precise spatial niches within solid tumors that determine the success or failure of anti-tumor immunity.

Recent work reveals that the TME is architecturally organized into specialized regions, such as tertiary lymphoid structures (TLS) associated with improved ICB response [12, 13], and “CRATER” niches identified in zebrafish as focal sites for CD8^+^ T cell engagement [14, 15]. These studies collectively establish spatial context as a decisive factor in immunotherapy outcomes [16, 17]. However, in human melanoma, a systematic mapping of pro-immune spatial domains-and the molecular regulators that define them-is strikingly absent. This knowledge gap severely limits the development of predictive biomarkers and rational combination therapies.

**Scheme 1:**
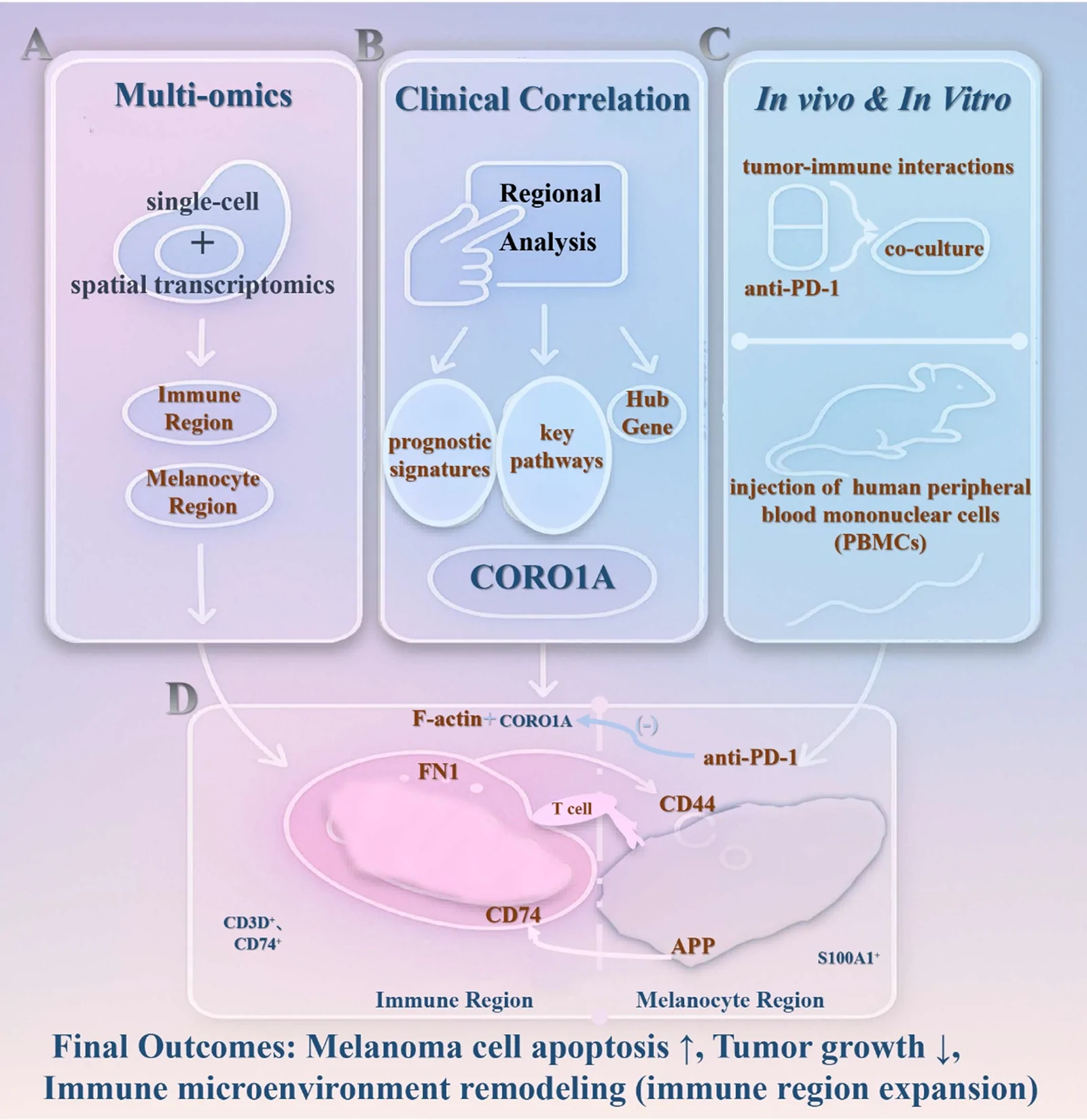
A schematic overview of the integrative multi-omics and functional validation workflow. This study delineates a multi-faceted research pipeline: **A**) Multi-omics Discovery: Integration of single-cell and spatial transcriptomics deconvolutes the tumor microenvironment and defines two key spatial domains—the Immune Region and Melanocyte Region. **B**) Clinical Correlation: Regional analysis identifies prognostic signatures and key inter-domain communication pathways (e.g., APP-CD74). Hub Gene Identification: Multi-omics convergence nominates CORO1A as a top candidate. **C**) Functional Validation: The role of CORO1A in modulating tumor-immune interactions and anti-PD-1 response is rigorously validated through in vitro co-culture assays and in vivo studies in a humanized mouse model. **D**) Integrated Hypothesis and Mechanism: Combined insights from A, B, and C propose a mechanistic model of how CORO1A regulates T cell function and immunotherapy response in the spatially organized melanoma microenvironment

Here, we delineate a multi-faceted research pipeline (Scheme 1) to dissect the architectural basis of anti-tumor immunity in cutaneous melanoma. First, through integrated single-cell and spatial transcriptomics (Scheme 1A), we deconvoluted the tumor microenvironment and identified two dominant spatial domains: an Immune region and a Melanocyte region. Building upon this spatial framework, clinical correlation and multi-omics convergence (Scheme 1B) not only demonstrated their prognostic power but also pinpointed inter-domain communication pathways (e.g., APP-CD74) and nominated *CORO1A* as a key regulator enriched within the immune niche. Subsequently, rigorous functional validation (Scheme 1C), employing in vitro co-culture assays and in vivo studies in a humanized mouse model, established that CORO1A modulates T cell-mediated killing and the response to anti-PD-1 therapy. Finally, by integrating these insights (Scheme 1D), we propose a mechanistic model of how CORO1A regulates T cell function within the spatially organized niche. Our study thus provides a spatially resolved blueprint of the melanoma TME and nominates CORO1A as a novel target for enhancing immunotherapy.

## Materials and methods

### Data acquisition and processing

The Skin Cutaneous Melanoma (SKCM) RNA-sequencing data (FPKM format) and corresponding clinical information were downloaded from The Cancer Genome Atlas (TCGA) portal (https://portal.gdc.cancer.gov/) using the TCGAbiolinks R package [18]. Samples lacking overall survival (OS) information were excluded, resulting in a final cohort of 98 primary solid tumor samples for prognostic analysis. Raw count data and additional clinical metadata were obtained from the UCSC Xena database [19]. The single-cell RNA sequencing (scRNA-seq) dataset GSE215121 [20] was retrieved from the Gene Expression Omnibus (GEO) database. This dataset, generated on the GPL20795 platform, encompasses three cutaneous melanoma samples from *Homo sapiens*. The two samples demonstrating superior data integration quality were selected for downstream analysis. Spatial transcriptomics data were acquired and processed using the R package BayesSpace [21]. A summary of all datasets used in this study is provided in Supplementary Table S1.

### Single-cell RNA-seq data analysis

scRNA-seq data from GSE215121 were processed using the Seurat package (v5.0) [22]. A Seurat object was created from the raw count matrix, retaining genes detected in a minimum of three cells and cells expressing at least 200 genes. Cells with a mitochondrial gene percentage exceeding 20% were filtered out to remove potential low-quality or stressed cells. Gene expression counts were normalized using the NormalizeData function. Principal Component Analysis (PCA) was performed on the variable features. Significant principal components (PCs) were determined using an elbow plot, and the top 30 PCs were selected for downstream analysis. Cell clusters were identified using the FindNeighbors and FindClusters functions at a resolution of 0.6, as determined by the clustree package. Dimensionality reduction and visualization were conducted using Uniform Manifold Approximation and Projection (UMAP).

### Cell type annotation and marker identification

Cell clusters were automatically annotated using the SingleR package [23] with the BlueprintEncodeData as the reference. Annotations were manually curated based on the expression of canonical marker genes. Dot plots were generated to visualize the expression of key marker genes across clusters. Differentially expressed genes (DEGs) for each cluster were identified using the FindAllMarkers function in Seurat. Genes with an absolute log_2_-fold change (|logFC|) >2, an adjusted *p*-value (adj.P) <0.05, and expressed in more than 50% of cells within a cluster were defined as marker genes for subsequent analysis.

### Gene set variation analysis (GSVA)

GSVA [24] was employed to assess pathway activity variations across cell types. Pseudo-bulk expression profiles were generated for each cell type using the AggregateExpression function in Seurat. The GSVA algorithm was then applied to these profiles using the HALLMARK gene sets from the msigdbr package. Gene sets with an adj.*p* < 0.05 were considered significantly enriched.

### Functional enrichment analysis

Functional enrichment analysis of Gene Ontology (GO) terms [25] (Biological Process, Molecular Function, Cellular Component) and Kyoto Encyclopedia of Genes and Genomes (KEGG) pathways [26] was performed using the clusterProfiler R package [27]. Terms with an adj.*p* < 0.05 and a False Discovery Rate (FDR) <0.25 were considered statistically significant, using the Benjamini-Hochberg (BH) correction method.

### Survival and differential expression analysis in TCGA database

The single-sample Gene Set Enrichment Analysis (ssGSEA) algorithm, implemented in the GSVA package, was used to calculate enrichment scores for specific cell types in the TCGA-SKCM bulk RNA-seq dataset. Kaplan-Meier (KM) survival analysis was performed based on the optimal cut-off of these scores to stratify patients into high and low groups. Differential expression analysis between these groups was conducted using the limma R package (v3.58.1) [28]. Genes with |logFC| >1 and adj.*p* < 0.05 were defined as DEGs. Volcano plots were visualized using ggplot2 (v3.4.4).

### Spatial transcriptomics analysis

Spatial transcriptomics data were imported and normalized using Seurat (v5.0). After performing PCA, the top 10 PCs were used for UMAP dimensionality reduction. Integration of scRNA-seq and spatial data for cell type deconvolution was achieved using two methods: Seurat’s FindTransferAnchors and TransferData functions, and the Robust Cell Type Decomposition (RCTD) method. Concordance between the two annotation approaches was evaluated by comparing spatial distribution patterns of major cell types and by inspecting the in situ expression of canonical marker genes, including S100A1 for melanocytes, CD74 for monocytes, and CD3D for T cells. Regional DEGs between spatially defined ‘Immune’ and ‘Melanocyte’ regions were subsequently identified using the FindMarkers function, with thresholds set at |logFC| >2 and adj.*p* < 0.001.

### Cell communication analysis

Cell communication was inferred using the CellChat R package [29], which integrates scRNA-seq expression data with a curated database of ligand-receptor interactions (CellPhoneDB.human). Significant interactions were identified based on communication probabilities and permutation tests. The number and strength of interactions were visualized using heatmaps and circle plots, respectively.

### Gene set scoring with AUCell

The AUCell package [30] was used to score the activity of predefined gene sets in single cells. The ‘Area Under the Curve’ (AUC) was calculated to determine the enrichment of specific gene signatures (e.g., Melanocyte or Monocyte marker genes) in the expression ranking of each cell. Results were visualized on UMAP and spatial maps. To reduce algorithm-specific interpretation, AUCell results were interpreted together with Seurat label transfer, RCTD deconvolution, GSVA, and ssGSEA-based analyses, rather than as a single standalone source of evidence.

### Prognostic model construction

Univariate Cox proportional hazards regression analysis was performed using the survival R package (v3.5–7) [31] to identify genes significantly associated with OS in the TCGA-SKCM cohort. Variables with a *p*-value < 0.05 from the univariate analysis were subsequently included in a stepwise multivariate Cox regression model using the step function from the R stats package. Model selection was based on the Akaike information criterion (AIC), with direction = ‘both’ to allow forward and backward selection. The final risk score was calculated as the sum of each selected gene’s expression multiplied by its corresponding Cox regression coefficient: risk score = sum(expri ×coefi). This workflow was used to identify the most parsimonious set of independent prognostic factors and construct a risk score.

### Somatic mutation analysis

The “Masked Somatic Mutation” data for SKCM were downloaded from the TCGA GDC portal and processed using VarScan software. Somatic mutation landscapes and oncogenic pathway alterations were analyzed and visualized using the maftools R package [32].

### Drug sensitivity prediction

Drug sensitivity (half-maximal inhibitory concentration, IC_50_) for common anticancer agents was predicted using the pRRophetic algorithm [33], which was trained on the Genomics of Drug Sensitivity in Cancer (GDSC) database (http://www.cancerRxgene.org). Differences in predicted IC_50_ values between patient groups were assessed using the Wilcoxon rank-sum test.

### Cell culture and reagents

The human melanoma cell line A375 and the Jurkat clone A3 T-cell line were purchased from Procell Life Science & Technology Co., Ltd. (China). Cells were maintained according to the supplier’s recommendations. Primary antibodies against CORO1A and PD-L1 were obtained from Abcam. All other chemical reagents and assay kits were sourced from Servicebio Biotechnology Co., Ltd. (China).

### In vitro functional assays

For co-culture experiments, Jurkat T cells were activated with CD4/CD8 antibodies (5 μg/ml) for 24 hours. Activated T cells were then co-cultured with A375 melanoma cells. Cell viability was assessed using the CCK-8 assay. Apoptosis was measured by flow cytometry. Gene expression was analyzed by quantitative real-time PCR (qPCR). Protein expression and localization were evaluated by Western Blot and immunofluorescence, respectively.

### In vivo xenograft studies

All animal experiments were approved by the Medical Ethics Committee of Hainan General Hospital (Approval No. Med-Eth-Re[2025]601) and conducted in accordance with institutional guidelines. For the studies, BALB/c nude mice (6–8 weeks old) were humanized by intraperitoneal injection of 5 × 10^6^ human peripheral blood mononuclear cells (PBMCs) obtained from healthy donors and validated for CD3+ T-cell purity before injection. Mice were monitored every three days for clinical signs of graft-versus-host disease (GVHD), including weight loss, ruffled fur, reduced mobility, and skin changes; no signs of GVHD were observed during the experiment.Two days PBMC transfer, 1 × 10^6^ A375 cells (either control shNC or *CORO1A*-knockdown shCORO1A) were subcutaneously injected into the left flank. Mice were divided into four groups (*n* = 4 per group): 1) shNC, 2) shCORO1A, 3) shNC + pembrolizumab, 4) shCORO1A + pembrolizumab. The *n* = 4 design was selected based on pilot effect-size estimates indicating an expected tumor inhibition difference of at least 40% (alpha = 0.05, power = 0.8), while also following the 3 R principle of animal ethics. Tumor volume was measured every three days. At the endpoint, tumors were harvested for qPCR and Western Blot analysis.

### Human tissue samples

A total of three pairs of human melanoma and adjacent normal tissue samples were collected. The use of these tissues was approved by the Medical Ethics Committee of Hainan General Hospital (Approval No. Med-Eth-Re[2025]601), and informed consent was obtained from all patients.

### Statistical analysis

All statistical analyses were performed in R (v4.2.0) or as indicated in specific method sections. Continuous variables are presented as mean ± standard deviation. Group comparisons for continuous data were conducted using the Wilcoxon rank-sum test. Correlations were assessed using Spearman’s rank correlation coefficient. An adjusted *p*-value (adj.P) <0.05 was considered statistically significant for all analyses, unless otherwise specified.

## Results

### Single-cell atlas of the cutaneous melanoma microenvironment

**Fig. 1:**
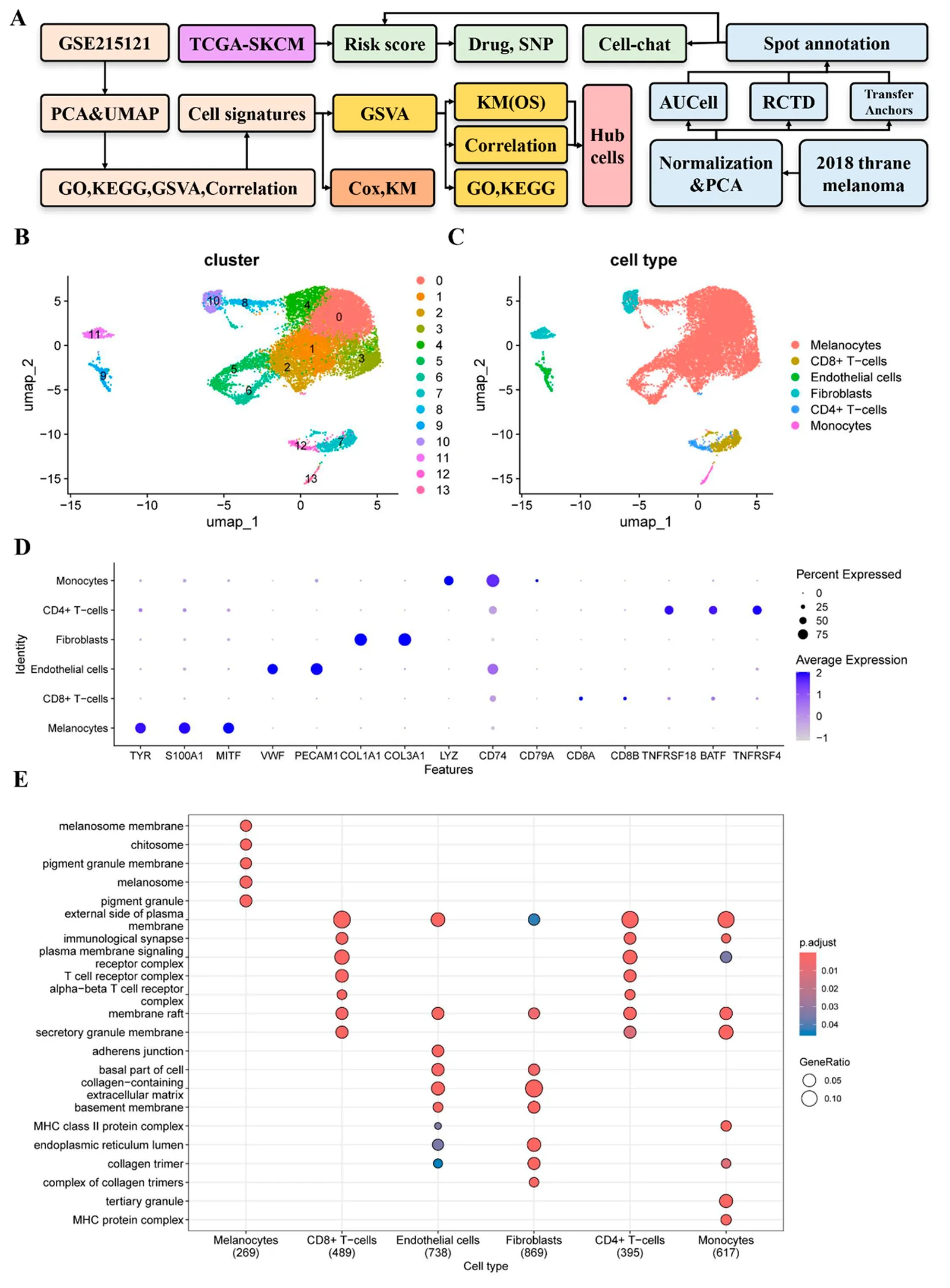
Single-cell transcriptomic profiling reveals the cellular architecture of cutaneous melanoma. (**A**) Flow chart. (**B**) UMAP visualization of 14 cell clusters identified in the scRNA-seq dataset (GSE215121). (**C**) UMAP plot colored by six major annotated cell types. (**D**) Dot plot showing the expression of key marker genes across the six cell types. Dot size represents the percentage of cells expressing the gene, and color intensity indicates the average expression level. (**E**) GO enrichment analysis (biological process and cellular Component) for marker genes of the indicated cell types.(abbreviations: PCA, principal Component Analysis; GSVA, gene Set Variation Analysis; KM, kaplan-Meier; OS, overall survival; SNP, single nucleotide polymorphism; UMAP, Uniform Manifold Approximation and Projection; GO, gene Ontology; KEGG, Kyoto encyclopedia of genes and Genomes; TCGA, the Cancer Genome Atlas; SKCM, skin cutaneous Melanoma; AUC, Area Under the Curve; RCTD, Robust cell type Decomposition)

To dissect the cellular complexity of cutaneous melanoma, we first performed single-cell RNA sequencing (scRNA-seq) analysis on the GSE215121 dataset (Fig. 1A). Uniform Manifold Approximation and Projection (UMAP) dimensionality reduction resolved the tumor ecosystem into 14 transcriptionally distinct clusters (Fig. 1B). Based on canonical marker gene expression using SingleR and manual curation, we annotated these clusters as six major cell types: Endothelial cells, Fibroblasts, Melanocytes, Monocytes, CD8^+^ T cells, and CD4^+^ T cells (Fig. 1C, D). Gene Ontology (GO) enrichment analysis confirmed the identity of these populations, with Melanocytes enriched in pigment-related pathways (e.g., melanosome), and T cells enriched in immune activation pathways (e.g., T cell receptor complex) (Fig. 1E). This analysis provides a foundational map of the cellular constituents within the melanoma tumor microenvironment (TME).

### Functional hallmarks and inter-cellular correlations within the tumor microenvironment

**Fig. 2:**
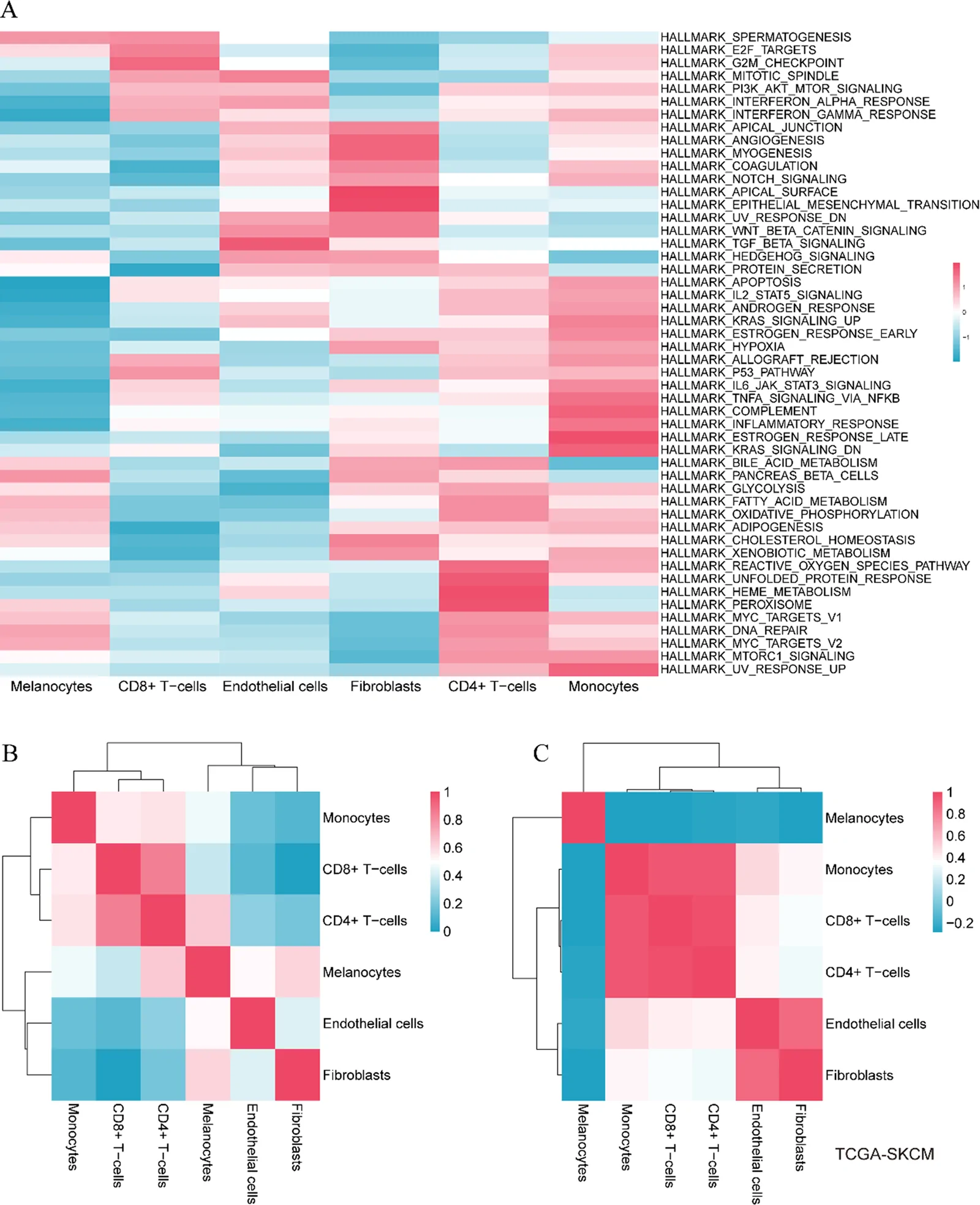
Functional and correlational landscape of melanoma cell populations. (**A**) Heatmap of GSVA enrichment scores for hallmark gene sets across the six major cell types. (**B**) Spearman correlation matrix of cell type abundances within the scRNA-seq dataset. (**C**) Spearman correlation matrix of cell type enrichment scores calculated by ssGSEA in the TCGA-SKCM cohort (Abbreviations: GSVA, Gene Set Variation Analysis; ssGSEA, single-sample Gene Set Enrichment Analysis; TCGA, The Cancer Genome Atlas; SKCM, Skin Cutaneous Melanoma)

We next sought to characterize the functional states of these cell populations. Gene Set Variation Analysis (GSVA) on hallmark gene sets reveals that Monocytes exhibit the highest enrichment scores for most pathways, while Melanocytes and CD8+ T cells show relatively quiescent transcriptional states (Fig. 2A). Correlation analysis of cell type abundances in the single-cell data demonstrated a strong positive correlation among immune cells (CD8^+^ T cells, CD4^+^ T cells, and Monocytes) (Fig. 2B). Strikingly, this co-occurrence pattern was recapitulated in the bulk transcriptomic data from the TCGA-SKCM cohort (Fig. 2C), suggesting a conserved, coordinated interplay within the immune compartment across different data modalities.

### Prognostic impact of immune cell infiltration and identification of key marker genes

**Fig. 3:**
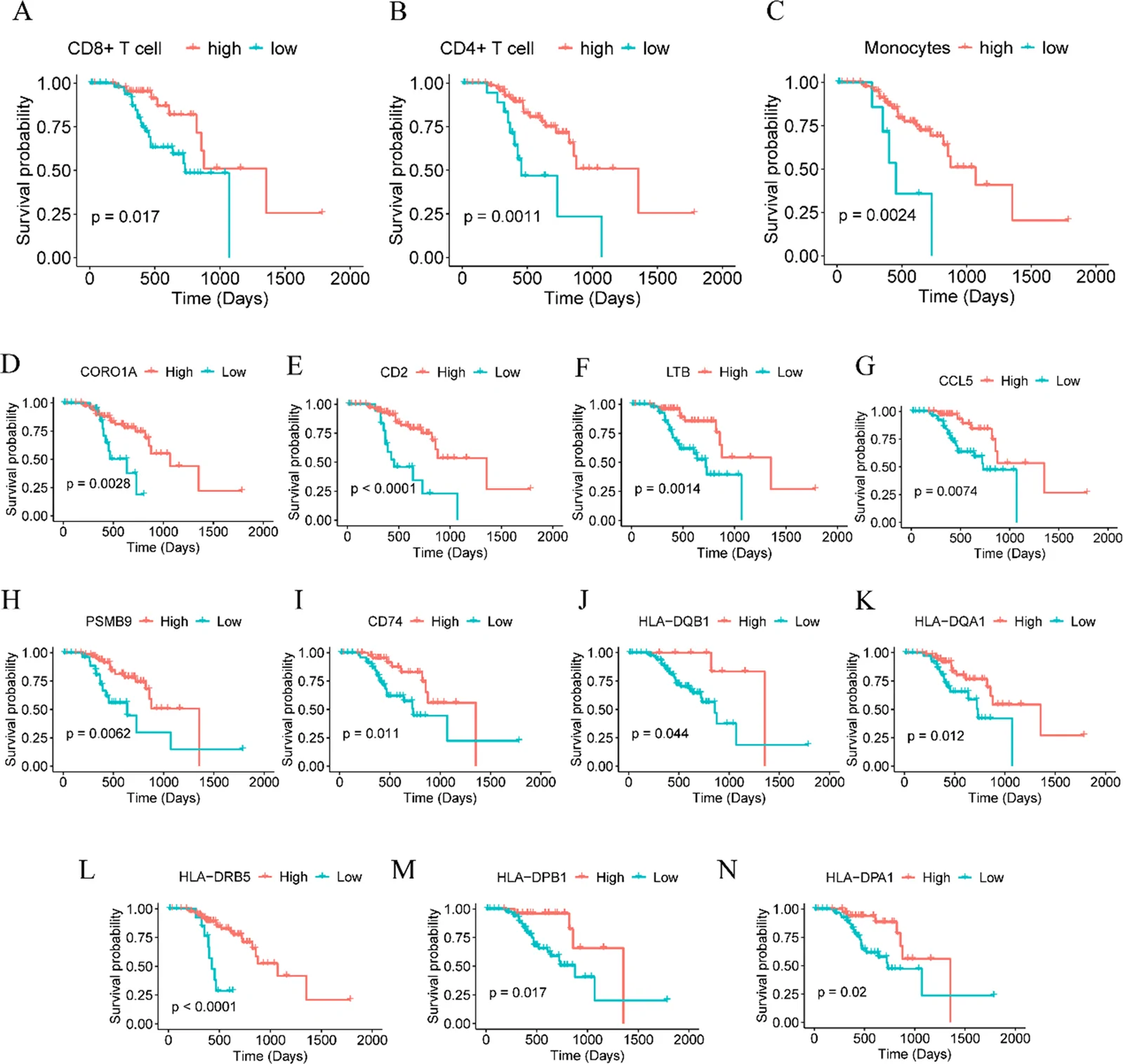
Immune cell abundance and specific marker genes are associated with patient survival. (**A-C**) Kaplan-Meier curves for overall survival (OS) in TCGA-SKCM patients stratified by high vs. low enrichment scores for (**A**) CD8^+^ T cells, (**B**) CD4^+^ T cells, and (**C**) Monocytes. (**D-N**) Kaplan-Meier curves for OS in TCGA-SKCM patients stratified by high vs. low expression of prognostic marker genes: (**D**) *CORO1A*, (**E**) *CD2*, (**F**) *LTB*, (**G**) *CCL5*, (**H**) *PSMB9*, (**I**) *CD74*, (**J**) *HLA-DQB1*, (**K**) *HLA-DQA1*, (**L**) *HLA-DRB5*, (**M**) *HLA-DPB1*, and (**N**) *HLA-DPA1* (Abbreviations: TCGA, The Cancer Genome Atlas; SKCM, Skin Cutaneous Melanoma; OS, Overall survival)

Leveraging the TCGA-SKCM cohort, we evaluated the clinical relevance of immune cell infiltration. Kaplan-Meier analysis revealed that high enrichment scores for CD8^+^ T cells, CD4^+^ T cells, and Monocytes were all significantly associated with improved overall survival (OS) (Fig. 3A–C). To identify the specific drivers of this favorable prognosis, we performed univariate Cox regression analysis on the marker genes of these cell types. This identified a panel of prognostic genes, including *CORO1A*, *CD2*, *LTB*, *CCL5*, and multiple MHC class II genes (*HLA-DQB1*, *HLA-DQA1*, *HLA-DRB5*, *HLA-DPB1*, *HLA-DPA1*), where high expression was associated with better patient outcomes (Fig. 3D–N).

### Spatial architecture defines immunologically distinct tumor niches

**Fig. 4:**
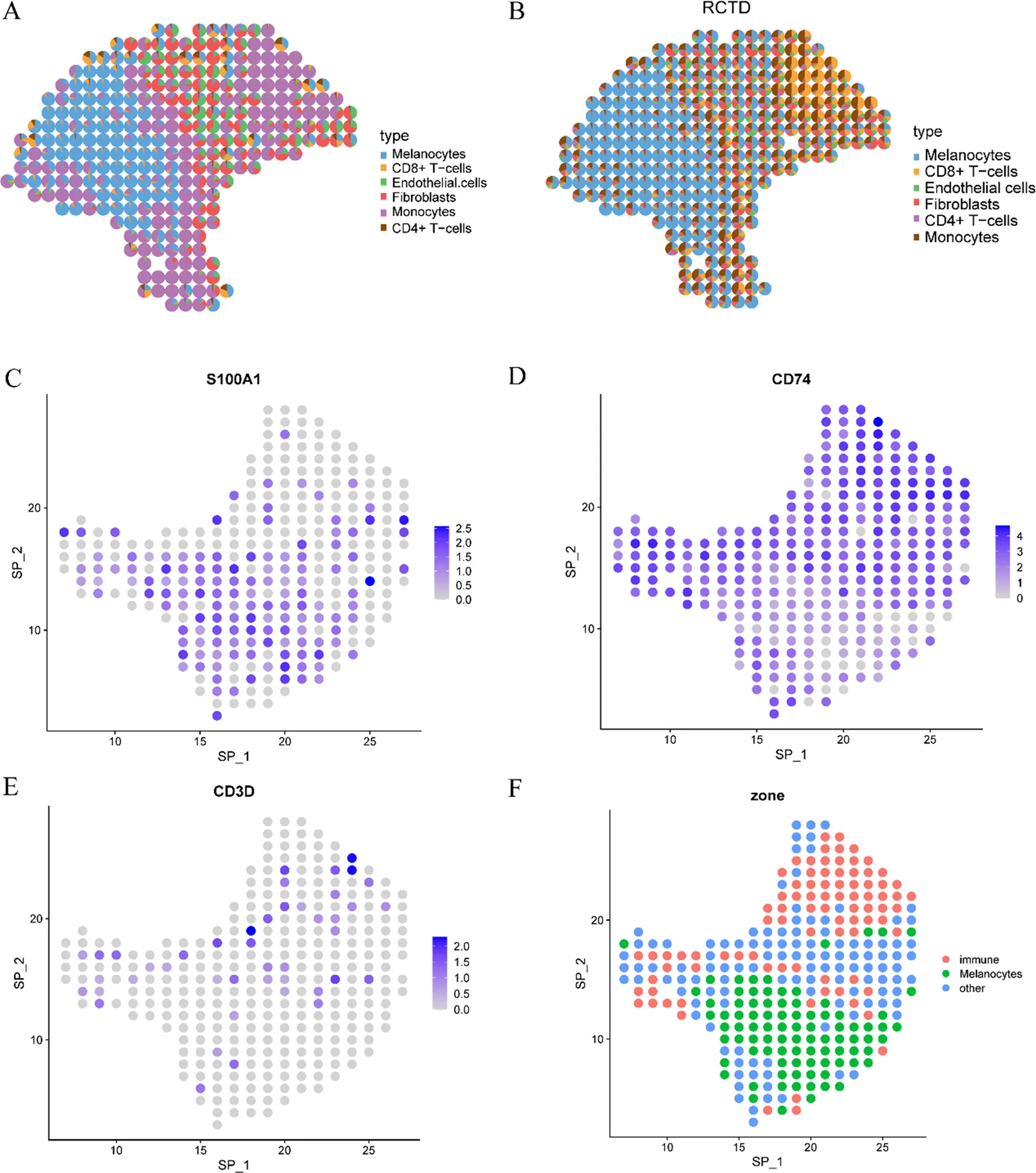
Spatial transcriptomics delineates immune and tumor cell niches. (**A, B**) Spatial distribution of cell types annotated by (**A**) Seurat’s integration and (**B**) RCTD. (**C-E**) Spatial expression patterns of representative marker genes: (**C**) *S100A1* (Melanocytes), (**D**) *CD74* (Monocytes), (**E**) *CD3D* (T cells). (**F**) definition of two major spatial domains: immune region and Melanocyte region. (Abbreviations: RCTD, Robust cell type Decomposition)

To transition from cellular dissociations to an intact tissue context, we analyzed spatial transcriptomics data. We integrated our scRNA-seq reference with the spatial data using two independent methods-Seurat’s label transfer and RCTD-both of which yielded concordant annotations, confirming the spatial distribution of the identified cell types (Fig. 4A, B). In situ expression mapping of key markers (S100A1 for Melanocytes, CD74 for Monocytes, CD3D for T cells) aligned with the spatial annotations and provided internal qualitative validation of the annotation accuracy (Fig. 4C–E). Based on this, we defined two major spatial domains: ‘Immune regions’, enriched for CD8+ T cells, CD4+ T cells, and Monocytes; and ‘Melanocyte regions’, dominated by tumor cells (Fig. 4F). This spatial partitioning provides a topological framework for the subsequent analysis of region-specific biology.

### Region-specific gene programs, inter-niche communication and clinical implications

**Fig. 5:**
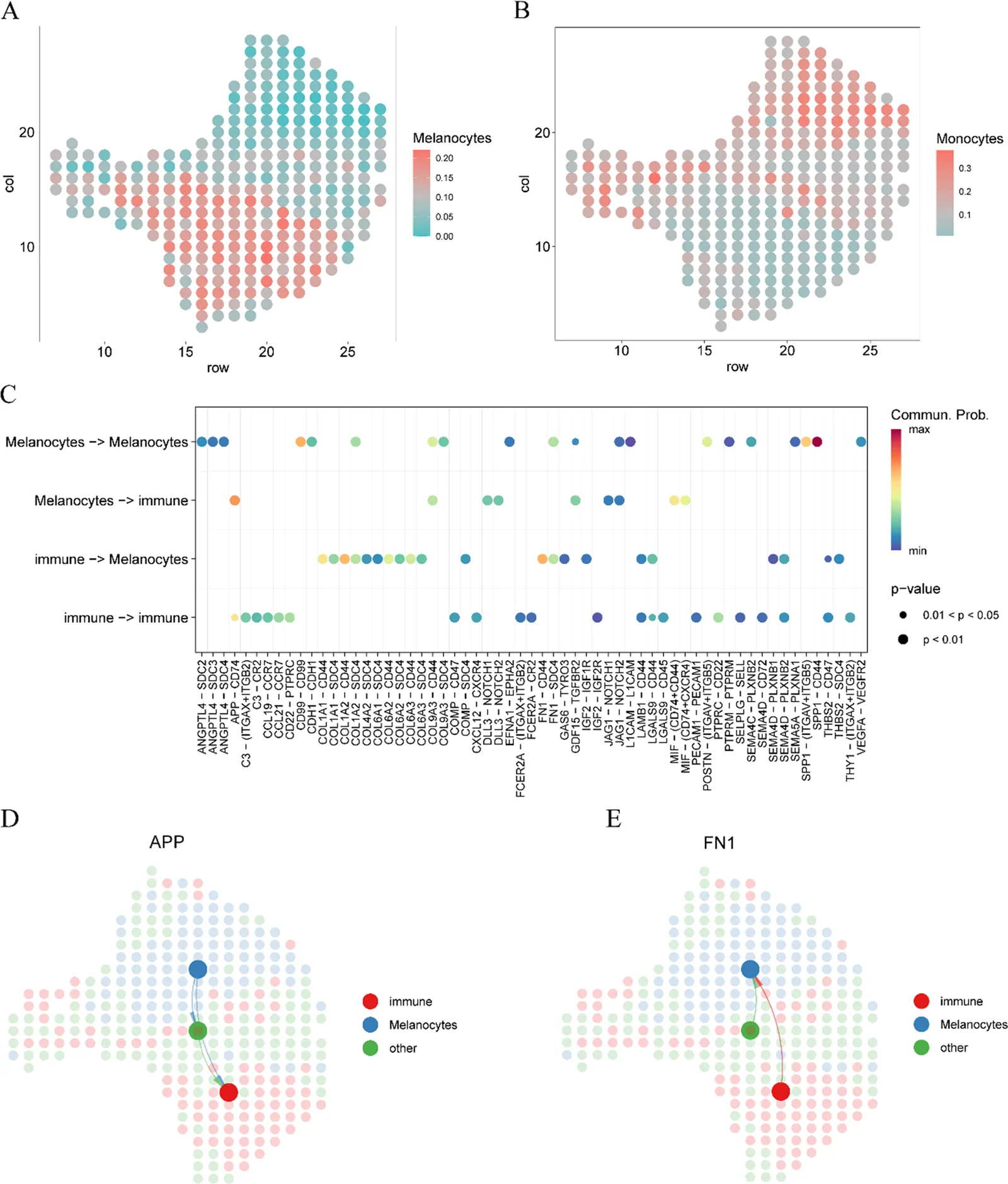
Spatial gene activity and inter-domain communication. (**A, B**) Spatial distribution of AUCell scores for (**A**) Melanocyte and (**B**) Monocyte gene signatures. (**C**) Bubble plot showing significant ligand-receptor interactions between the Melanocyte and immune regions. Dot size indicates statistical significance, and color indicates communication probability. (**D, E**) Circle plots depicting the interaction networks for the (**D**) APP-CD74 and (**E**) FN1–CD44 pathways. (Abbreviation: AUC, Area Under the Curve)

We then characterized the molecular features of these spatial domains. AUCell scoring confirmed that the gene programs of Melanocytes and Monocytes were highly active in their respective spatial regions (Fig. 5A, B). These AUCell patterns were consistent with Seurat label transfer and RCTD deconvolution, and the broader functional interpretation was also supported by GSVA and ssGSEA analyses, indicating that the definition of spatial regions did not rely on a single gene-set scoring algorithm. To understand how these regions interact, we performed cell-cell communication analysis. This revealed that the APP signaling pathway (APP-CD74) was the most prominent outgoing signal from the Melanocyte region to the Immune region, while the FN1 pathway (FN1–CD44) was a key signal from the Immune region to the Melanocyte region (Fig. 5C). The interaction networks for these two pathways are detailed (Fig. 5D, E), highlighting potential mechanisms of cross-talk between tumor and immune compartments.

To further assess the clinical utility of our spatial findings, we investigated whether these spatial domains were linked to distinct prognostic signatures, genomic features, and therapeutic opportunities. First, we identified genes differentially expressed between the Melanocyte and Immune regions. Univariate Cox analysis identified prognostic genes specific to each region, including ANGPT2, DUSP4, KDR in the Melanocyte region, and CD74, CORO1A, CD2 in the Immune region (Supplementary Fig. S1A, B). We then constructed two separate risk scores using stepwise Cox regression. Both the Melanocyte region risk score and the Immune region risk score significantly stratified patients into high-risk and low-risk groups with distinct overall survival outcomes (Supplementary Fig. S1C, D), underscoring the prognostic power embedded in the spatial organization of the TME.

Next, we explored the association between these spatial risk groups and underlying genomic alterations. Mutational analysis revealed that high- and low-risk groups exhibited different spectra of somatic mutations (e.g., in TENM4, MYH2, BRAF; Supplementary Fig. S2A and S3A) and were associated with alterations in key signaling pathways such as RTK-RAS and NOTCH (Supplementary Fig. S2B, C and S3B, C). Interrogation of the DGIdb database identified potentially actionable genes in each group (Supplementary Fig. S2D, E and S3D, E). Finally, drug sensitivity analysis predicted that patients in the high-risk groups (both Melanocyte and Immune) were significantly less sensitive to a range of targeted and chemotherapeutic agents (Supplementary Fig. S4, S5), highlighting the potential for spatial domain classification to guide personalized therapy.

### CORO1A as a key mediator of melanoma cell-immune interactions

**Fig. 6:**
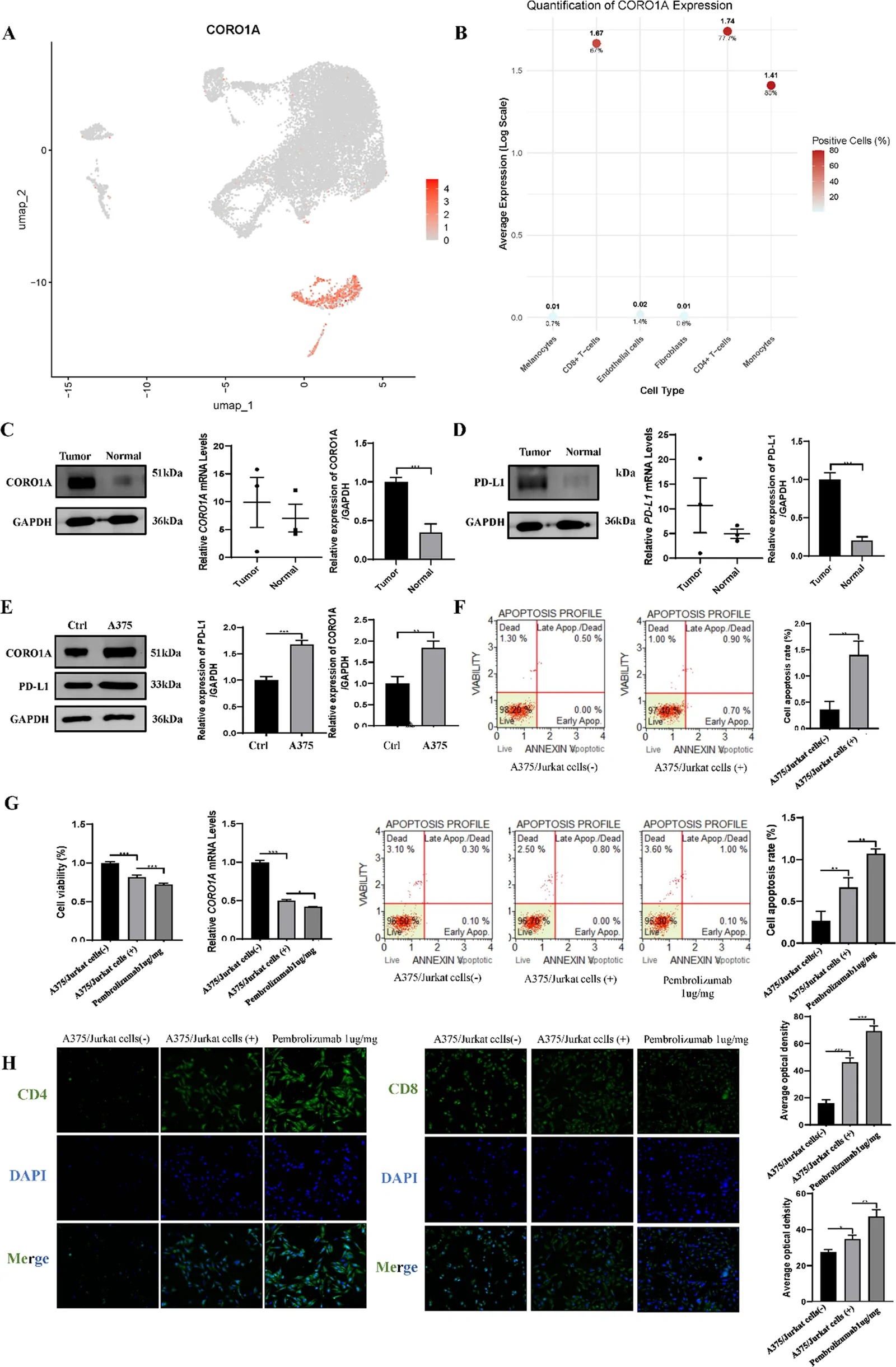
CORO1A modulates T cell-mediated killing and response to anti-PD-1 in vitro. (**A**) Quantitative single-cell analysis of CORO1A expression across annotated cell populations. (**B**) CORO1A-positive cell proportions and average expression levels in the major cell populations. (**C, D**) CORO1A and PD-L1 are preliminarily upregulated in human melanoma tissues. (**E**) CORO1A and PD-L1 expression in melanoma cell lines. (**F**) Co-culture with activated T cells reduces melanoma cell viability and induces apoptosis. (**G**) pembrolizumab enhances T cell-mediated cytotoxicity in a dose-dependent manner and downregulates CORO1A expression in the co-culture system. (**H**) immunofluorescence analysis of T cell infiltration and tumor cell death after anti-PD-1 treatment

The integration of scRNA-seq, spatial transcriptomics, and TCGA bulk RNA-seq data converged to point to *CORO1A*, a prognostic gene enriched in the Immune region, as a candidate key regulator. To clarify its cellular source, we quantified *CORO1A* expression across all annotated cell populations in the scRNA-seq data. *CORO1A* showed a strong immune-cell-enriched pattern, with high average expression in CD4+ T cells (1.74; 77.7% positive cells), CD8+ T cells (1.67; 67.0% positive cells), and Monocytes (1.41; 80.0% positive cells). In contrast, *CORO1A* was nearly absent from non-immune populations, including melanocytes, endothelial cells, and fibroblasts, with average expression values ≤ 0.02 and positive-cell proportions < 1.5%. These data indicate that *CORO1A* is primarily derived from T lymphocytes and monocytes in the single-cell atlas, while leaving open the possibility of low-level or context-dependent tumor-cell expression (Fig. 6A, B). We next performed preliminary tissue validation and observed that *CORO1A* and PD-L1 protein levels were elevated in three paired human melanoma tissues compared to adjacent normal skin (Fig. 6C, D). Because this cohort was small, these results should be interpreted as preliminary validation rather than definitive clinical confirmation. In vitro, we confirmed that the A375 melanoma cell line expressed both *CORO1A* and PD-L1 (Fig. 6E). Co-culture of A375 cells with pre-activated Jurkat T cells led to a significant reduction in melanoma cell viability and an increase in apoptosis, demonstrating T cell-mediated killing (Fig. 6F). Treatment with the PD-1 inhibitor pembrolizumab enhanced this cytotoxic effect in a dose-dependent manner. In the co-culture system, pembrolizumab treatment not only augmented tumor cell killing but also downregulated *CORO1A* expression (Fig. 6G). To further delineate the immunomodulatory effect, we performed immunofluorescence and live/dead staining. We found that pembrolizumab treatment markedly increased the infiltration of both CD4+ and CD8+ T cells into the tumor cell clusters (Fig. 6H), concomitant with enhanced tumor cell death. These data collectively suggest a functional link between immune checkpoint blockade and the *CORO1A* pathway, and demonstrate that anti-PD-1 therapy potentiates T cell recruitment and cytotoxicity.

### CORO1A knockdown potentiates anti-tumor immunity and response to anti-PD-1 in vivo

**Fig. 7:**
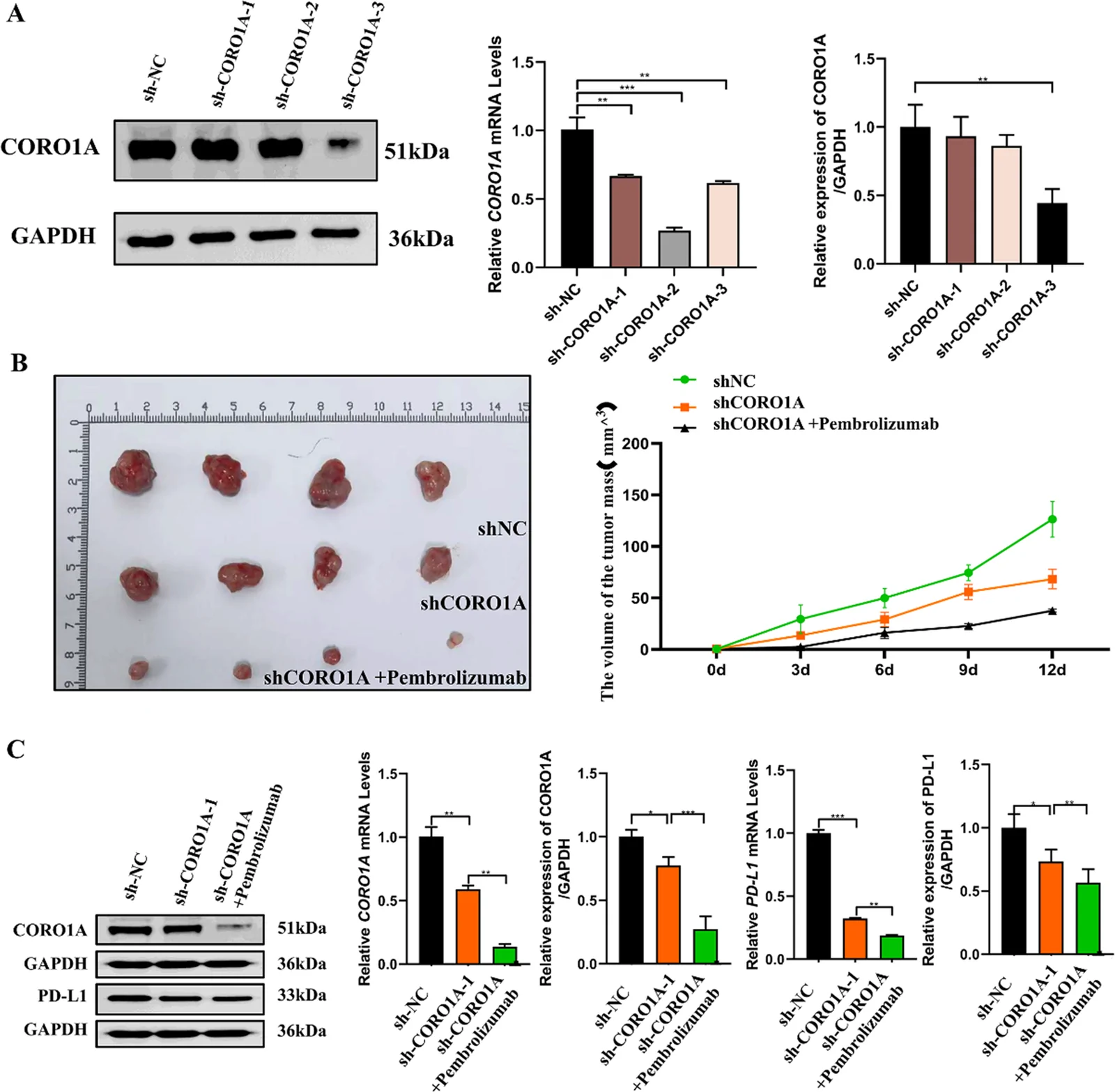
CORO1A depletion synergizes with anti-PD-1 therapy to suppress tumor growth in vivo. (**A**) Validation of *CORO1A* knockdown efficiency in A375 cells. (**B**) Tumor growth curves in humanized mouse models across four treatment groups: shNC, shCORO1A, shNC + pembrolizumab, shCORO1A + pembrolizumab. (**C**) analysis of *CORO1A* and PD-L1 expression in resected tumors from each group

To establish the role of CORO1A in vivo, we employed a humanized mouse model. We stably knocked down CORO1A in A375 cells (Fig. 7A) and engrafted them into immunodeficient mice reconstituted with human PBMCs. Compared to the control group, mice bearing CORO1A-knockdown tumors exhibited significantly suppressed tumor growth (Fig. 7B). Furthermore, the combination of CORO1A knockdown and pembrolizumab treatment resulted in the most potent tumor inhibition (Fig. 7B). Analysis of excised tumors confirmed decreased CORO1A and PD-L1 expression in the knockdown groups (Fig. 7C). Given the modest group size, these in vivo data support a proof-of-concept interaction between CORO1A depletion and anti-PD-1 treatment, but larger studies with detailed immune profiling will be required to fully establish therapeutic synergy and mechanism.

## Discussion

Cutaneous melanoma is a highly aggressive malignancy with a propensity for metastasis and treatment resistance. While immune checkpoint blockade (ICB) has markedly improved outcomes for a subset of patients, primary and acquired resistance remain prevalent [34, 35], underscoring a critical need to decipher the determinants of immune response within the tumor microenvironment (TME) [36]. Recent work on melanoma immune microenvironment biology, microbiota-related immune regulation, cytokine-mediated tumor sensitization, and TIME-associated prognostic signatures further emphasizes the need to integrate spatial organization with molecular and immune-context features [37–40]. A key limitation is our incomplete understanding our incomplete understanding of how the spatial organization of the TME influences the functional capacity of tumor-infiltrating lymphocytes.

By integrating multi-omics data, we have decoded this spatial architecture, defining two predominant and prognostically significant domains: an Immune region and a Melanocyte region. Furthermore, through convergent bioinformatic and functional analyses, we identify *CORO1A* as a key gene enriched within the immune niche, which plays a functional role in modulating T cell-mediated cytotoxicity and response to anti-PD-1 therapy [41]. Our findings provide a spatial and molecular rationale for immunotherapy success and failure, suggesting novel avenues for therapeutic intervention.

Our discovery of spatially segregated immune and tumor cell niches not only resonates with but also expands upon recent concepts of specialized functional units within the TME. Ludin et al. recently identified “CRATERs” in zebrafish melanoma as stromal-derived pockets where CD8^+^ T cells recognize and engage tumor cells [42]. Similarly, our defined Immune region represents a physical compartment where cytotoxic T cells, helper T cells, and antigen-presenting monocytes co-localize. While CRATERs are described as discrete, focal structures at the stroma-tumor interface, the Immune region we identify in human tumors manifests as a larger, contiguous area. This distinction may reflect an advanced stage of immune consolidation where initial, focal T cell engagements (akin to CRATERs) expand and coalesce into a broader functional domain during a productive anti-tumor response. Alternatively, the difference could arise from divergent methodological and biological scales—high-resolution live imaging in zebrafish captures nascent, microscale interactions, whereas spatial transcriptomics in human tissues reveals stabilized, tissue-level architecture [8]. Despite these differences, both structures underscore the fundamental principle that effective anti-tumor immunity is topologically organized within privileged spatial niches, rather than being diffusely distributed.

The cell-cell communication analysis revealed APP-CD74 and FN1–CD44 as key ligand-receptor axes bridging the Melanocyte and Immune regions. The APP-CD74 interaction, identified as a major outgoing signal from the Melanocyte region, may represent a novel immunosuppressive mechanism in melanoma. Beyond promoting tumor survival, tumor-derived APP can also function as a “don’t eat me” signal by engaging CD74 on infiltrating myeloid cells, potentially inhibiting phagocytosis and skewing macrophages toward an M2-like, pro-tumor phenotype, which aligns with the immunosuppressive character of this niche [43, 44]. Conversely, FN1 from the Immune region engaging CD44 on tumor cells extends beyond influencing adhesion and migration. This interaction likely contributes to immune microenvironment remodeling by promoting extracellular matrix deposition that forms a physical barrier, potentially trapping CD44-expressing immune cells and restricting their cytotoxic penetration into the tumor core, while also offering a targetable axis for drug delivery strategies [45]. This bidirectional cross-talk paints a dynamic picture of a co-evolved niche where tumor and immune cells continuously remodel their microenvironment through spatially organized molecular dialogues that reinforce immune suppression.

A central finding of our work is the functional characterization of CORO1A, an actin-binding protein predominantly expressed in hematopoietic cells and essential for cytoskeletal dynamics in T cells [46]. While its role in general T cell physiology is known, its specific function within the immunosuppressive tumor microenvironment of melanoma has been undefined. Our single-cell quantification indicates that CORO1A is mainly enriched in CD4+ T cells, CD8+ T cells, and monocytes, supporting an immune-cell-centered role in cytoskeletal dynamics, immune synapse formation, and cytotoxic function. At the same time, the A375 experiments suggest that CORO1A may also have a non-canonical, tumor-intrinsic role in melanoma cells, because CORO1A knockdown reduced tumor growth and PD-L1 expression. Thus, the current evidence supports two distinguishable but potentially connected compartments: an immune-cell function related to T-cell and myeloid-cell biology, and a tumor-cell function related to melanoma cell growth and immune-evasion signaling. Importantly, the present knockdown strategy cannot fully separate cell-type-specific contributions, and future studies using cell-type-selective perturbation systems will be required to determine whether the dominant therapeutic effect arises from immune cells, tumor cells, or both. These findings collectively reposition CORO1A from a mere marker to a functional modulator of antitumor immunity within the spatially organized tumor niche [47].

The clinical translatability of our findings is supported by the prognostic performance of the spatial domain-derived risk scores, although these models should currently be viewed as exploratory tools rather than clinically validated decision instruments. The TCGA-SKCM analysis included 98 primary solid tumor samples, and the univariate Cox plus stepwise multivariate Cox workflow may be vulnerable to overfitting in the absence of independent validation. Therefore, the risk scores are best interpreted as statistical screening tools that support the biological relevance of spatially derived gene programs, while the core conclusion regarding CORO1A is further supported by functional experiments. Future validation in larger independent melanoma cohorts, ideally with external immunotherapy-response datasets, elastic-net or LASSO-Cox modeling, bootstrap optimism correction, time-dependent ROC curves, calibration analysis, and decision-curve analysis will be necessary before clinical deployment [3, 48]. The fact that both the Melanocyte and Immune region risk models significantly stratified patient survival highlights that both tumor-intrinsic programs and the surrounding immune contexture are independent yet complementary determinants of outcome. Moreover, the distinct mutational landscapes and drug vulnerability profiles associated with these risk groups offer a potential roadmap for personalized therapeutic strategies. For instance, patients in the high-risk Melanocyte group might benefit more from targeting the specific signaling pathways (e.g., RTK-RAS) identified in our mutation analysis, while high-risk Immune group patients might be candidates for agents that disrupt immunosuppressive axes such as APP-CD74 [49, 50].

An intriguing and unexpected finding from our functional validation is the expression of CORO1A in melanoma cells (e.g., A375) and the impact of its knockdown on tumor growth and PD-L1 expression in vivo. This result should be interpreted separately from the dominant immune-cell-enriched expression pattern observed in the single-cell atlas. In immune cells, especially T cells, CORO1A likely contributes to cytoskeletal remodeling, immune synapse formation, migration, and cytotoxic effector function. In melanoma cells, however, CORO1A may exert a non-canonical, tumor-intrinsic function. Based on its established molecular function, one plausible hypothesis is that CORO1A regulates cytoskeletal organization, cell motility, or intracellular signaling pathways in melanoma cells, akin to its role in immune cells. This could influence processes such as local invasion or the formation of specific spatial structures [11, 51]. Furthermore, its knockdown-associated reduction in tumor PD-L1 levels hints at a possible link between CORO1A and immune checkpoint molecule regulation, although the precise mechanistic connection remains to be elucidated [52, 53]. This observation expands the potential therapeutic relevance of CORO1A beyond modulating T-cell function to possibly directly targeting tumor cell properties that facilitate immune evasion [54, 55].

Our study has several limitations. First, the spatial transcriptomics data, while informative, lack single-cell resolution, and the inferences about cellular communication require further experimental validation. Although Seurat label transfer, RCTD deconvolution, and marker-gene expression showed concordant spatial patterns, serial IHC sections, multiplex immunofluorescence, or spatial proteomics from the same tissue sections were not available for external quantitative validation. Second, the single-cell reference atlas excluded one low-quality sample based on objective QC metrics, but this selection may still introduce bias; future sensitivity analyses including larger sample numbers will be valuable. Third, the human tissue validation included only three paired melanoma and adjacent normal samples, so the observed upregulation of CORO1A and PD-L1 should be considered preliminary and requires confirmation in larger independent cohorts or tissue microarrays with quantitative IHC or multiplex immunofluorescence. Fourth, the Jurkat-A375 co-culture system is a proof-of-concept model and does not fully reproduce primary exhausted T-cell biology; Jurkat cells may have limited PD-1-dependent cytotoxic features, and only one melanoma cell line was used for knockdown experiments. Future studies should use primary patient-derived T cells, additional melanoma cell lines, and flow-cytometry-based assays of PD-1, CD69, IFN-gamma, GZMB, PRF1, TNF-alpha, and tumor-cell-specific apoptosis. Fifth, the humanized mouse study used a modest *n* = 4 per group and lacked detailed immune profiling because harvested tumors were consumed for qPCR and Western Blot analysis. Future in vivo experiments should include larger cohorts and assessment of human CD45+ engraftment, intratumoral CD8+ T cells, CD4+ T cells, macrophage infiltration, Ki-67, cleaved caspase-3, GZMB, IFN-gamma, and PD-1/PD-L1 expression. In addition, pembrolizumab primarily targets human PD-1-positive T cells in this humanized model and is not recognized by the mouse immune system, which may limit the extent to which host-drug interactions recapitulate the clinical setting. Finally, the prognostic models remain exploratory and require independent validation with penalized regression, bootstrap correction, and clinically interpretable performance analyses before they can guide patient stratification.

## Conclusion

In conclusion, our work provides a high-resolution, spatially aware blueprint of the cutaneous melanoma microenvironment. We move beyond a simple catalog of cell types to define functional anatomic units that dictate disease progression and therapy response. By identifying and validating *CORO1A* as a crucial node within the pro-immune spatial niche, we not only advance our fundamental understanding of tumor-immune interactions but also nominate a promising candidate for developing novel combination immunotherapies. Future efforts aimed at therapeutically co-opting these spatial domains and their key regulators, like *CORO1A*, hold significant potential to improve outcomes for melanoma patients.

## Electronic Supplementary Material

Below is the link to the electronic supplementary material.

**Supplementary Material 1: Figure S1**. Prognostic Risk Models Derived from Spatial Domains.(A, B) Forest plots of univariate Cox regression analysis for genes highly expressed in the (A) Melanocyte region and (B) Immune region. (C, D) Kaplan-Meier survival curves for patients stratified by high vs. low (C) Melanocyte region risk score and (D) Immune region risk score

**Supplementary Material 2: Figure S2**. Somatic Mutations and Druggable Alterations in Melanocyte Region Risk Groups.(A) Oncoplot of the top mutated genes in the high and low Melanocyte region risk groups. (B, C) Altered signaling pathways in the (B) high-risk and (C) low-risk groups. (D, E) Classification of potentially druggable genes in the (D) high-risk and (E) low-risk groups

**Supplementary Material 3: Figure S3**. Somatic mutation analysis in high and low grouping of immune regions

**Supplementary Material 4: Figure S4**. Melanocytes regional grouping drugs sensitivity

**Supplementary Material 5: Figure S5**. High and low groups of drug sensitivity in immune regions

**Supplementary Material 6: Table S1**. Data information

## Data Availability

The datasets generated and/or analyzed during the current study are available in the Gene Expression Omnibus (GEO) repository (GSE215121) and from The Cancer Genome Atlas (TCGA) portal (https://portal.gdc.cancer.gov/). Other data supporting the findings of this study are available from the corresponding author upon reasonable request.

## Abbreviations

ICB: Immune checkpoint blockade
TME: Tumor microenvironment
scRNA-seq: Single-cell RNA sequencing
GEO: Gene Expression Omnibus
TCGA: The Cancer Genome Atlas
OS: Overall survival
UMAP: Uniform Manifold Approximation and Projection
GO: Gene Ontology
KEGG: Kyoto Encyclopedia of Genes and Genomes
GSVA: Gene Set Variation Analysis
ssGSEA: Single-sample Gene Set Enrichment Analysis
RCTD: Robust Cell Type Decomposition
AUC: Area Under the Curve
PBMCs: Peripheral blood mononuclear cells
qPCR: Quantitative real-time PCR

## Declarations

### Author information

SZ, HW, JW, PF, YL, TM, and LL are affiliated with the Hainan General Hospital, Hainan Medical University.

### Generative AI and AI-assisted

Generative AI and AI-assisted technologies were NOT used in the preparation of this work.
