# Supplementary material for: Spatial architecture of the melanoma immune niche reveals CORO1A as a functional hub for T cell cytotoxicity and immunotherapy synergy
Source: J Transl Med. 2026 Jun 23;24:1159. doi: 10.1186/s12967-026-08435-0 (PMC13555885; doi:10.1186/s12967-026-08435-0)
Supplement: Supplementary file 1 [file 12967_2026_8435_MOESM6_ESM.docx]

**Table S1 data Information**

|  | TCGA-SKCM | 2018.Thrane.melanoma | GSE215121 |
| --- | --- | --- | --- |
| Platform | TCGA | Spatial Transcriptomics v1.0 | GPL20795 |
| Species | Homo sapiens | Homo sapiens | Homo sapiens |
| Tissue | skin squamous cell carcinoma | skin squamous cell carcinoma | skin squamous cell carcinoma |
| Samples in SKCM Group | 98 | 1 | 2 |
